# Cystic fibrosis telemedicine in the era of COVID-19

**DOI:** 10.1093/jamiaopen/ooac005

**Published:** 2022-02-09

**Authors:** Elika J Rad, Alicia A Mirza, Laveena Chhatwani, Natasha Purington, Paul K Mohabir

**Affiliations:** 1 Chest Clinic, Stanford Health Care, Stanford, California, USA; 2 Department of Medicine, Stanford University School of Medicine, Stanford, California, USA; 3 Department of Medicine, Stanford University, Stanford, California, USA

**Keywords:** telemedicine, cystic fibrosis, pulmonary disease, pandemic, coronavirus

## Abstract

The coronavirus disease 2019 pandemic has resulted in large-scale changes to incorporate telemedicine for the delivery of care. People with cystic fibrosis (CF) have care considerations that pose challenges to telemedicine; they include frequent visits for pulmonary disease progression, medication management, and evaluation by a multidisciplinary team of providers. We share our center’s experience with video visits replacing in-person clinic evaluation, using quality improvement strategies to create a replicable workflow. Key considerations include incorporation of the multidisciplinary team into the visit, limitations of remote delivery of care, as well as patient and staff perceptions of this care model. Results revealed that video visits were convenient, efficacious, and comparable to in-person visits, with interest for its continued incorporation into the traditional CF care model.

## Lay Summary

The coronavirus disease 2019 pandemic has resulted in the need to incorporate telemedicine as a form of patient care. Cystic fibrosis (CF) is a genetic disease affecting the lungs and multiple other organs. CF patients require frequent clinic visits for disease monitoring and medication management provided by a team of physicians, respiratory therapists, nurses, dietitians, and social workers. We share our CF center’s experience with video visits replacing in-person clinic evaluation during the pandemic using a patient and staff survey. Our results showed the telemedicine care model was convenient, efficacious, and similar to in-person visits, with interest for its continued beyond the pandemic.

## INTRODUCTION

To ensure patient and personnel safety during the coronavirus disease 2019 (COVID-19) pandemic, healthcare systems were forced to enact strict infection control measures, including the cancellation of clinics. The significant reduction of in-person patient evaluation propelled telemedicine to the forefront of healthcare. Swift incorporation of this modality into the Adult Cystic Fibrosis (CF) clinic at a large academic center became essential for providing continuity of care and chronic disease management. However, the standard quarterly in-person evaluations with onsite testing and multidisciplinary evaluations posed challenges. In this article, we describe the transition to our algorithmic model for telehealth. By way of survey, we evaluated patient and staff perceptions of this new clinic structure.

## BACKGROUND

CF is an autosomal recessive genetic illness caused by mutations in the CF transmembrane regulator (CFTR) protein, resulting in disruption of CFTR-mediated chloride transport in widely distributed epithelial surfaces. As a result, most individuals with CF develop pulmonary disease characterized by progressive and severe bronchiectasis as well as multisystem manifestations to include sinusitis, pancreatic insufficiency, diabetes, intestinal obstruction, malnutrition, liver disease, and infertility, leading to early morbidity and mortality. Though the prevalence and incidence of CF vary by ethnic groups, the prevalence is highest in white persons of European origin.^1^ The median predicted survival has improved drastically for patients with CF from 32 years between 1995 and 1999 to 46 years for those born between 2015 and 2019.^2^ This is due to several factors including improvement in inhaled therapies for symptom control, early treatment of pulmonary exacerbations, aggressive nutritional management, the multidisciplinary care model, newborn screening, and lung transplantation.^3^^,^^4^ The advent of novel therapies such as CFTR protein modulators is predicted to further bolster the survival statistics.

Maintenance of optimal respiratory health in this population requires close clinical monitoring at an accredited CF center, with the recommendation for minimum quarterly visits as standard care.^5^ Older epidemiologic data further demonstrate that CF care centers with the best outcomes offer more frequent monitoring of clinical status by the availability of clinic visits, lung function tracking via spirometry, more frequent respiratory culture surveillance, and longer and more frequent courses of IV antibiotics.^6^ In the era of a pandemic, although minimizing contact and exposure to health centers is a key step in mitigating COVID-19 infection risk, alternate methods of patient assessment and access to healthcare are imperative to maintaining optimal pulmonary health in this vulnerable population. This is where telehealth self-monitoring tools such as home spirometers and pulse oximetry data, home respiratory culture collection, as well as virtual clinic visits can play a crucial role.

To accommodate the surge of patients covered under the 2010 Affordable Care Act, remote delivery of services using telehealth was considered. Specifically, telemedicine or mobile health using synchronous video visits alleviated barriers such as cost, distance to clinics, and availability of specialized care and appointments.^7^ Initially, interstate reimbursement variability posed significant barriers to meaningful implementation. Although Medicare began reimbursement for telehealth in 1997, most private insurance companies lagged. Ultimately, parity laws mandated payment for telehealth akin to in-person visits. This resulted in partial reimbursement as well increased utilization of telehealth visits from 2010 to 2015.^8^ Privacy changes under the Health Insurance Portability and Accountability Act (HIPAA) allowed for secured mobile device use with electronic health record (EHR) portals.^7^ The COVID-19 pandemic further lifted telemedicine regulatory barriers to include care delivery across state lines.^9^

A few studies have looked at the impact of telemedicine on chronic obstructive pulmonary disease outcomes, demonstrating reduced hospital admissions and fewer sick days using self-monitoring devices.^10–12^ There have not been robust studies of telemedicine in CF. One meta-analysis concluded insufficient evidence regarding the benefits of this care model.^13^ Study limitations included study group and data heterogeneity, underpowered trials, and lack of randomization.^13^^,^^14^ A small study of adult CF patients in Western Australia demonstrated improved clinic attendance and higher patient satisfaction scores with availability of either video or in-person visits.^15^ Another small feasibility study demonstrated improved CF patient satisfaction using social video messaging applications.^16^

## METHODS

**Figure 1.**
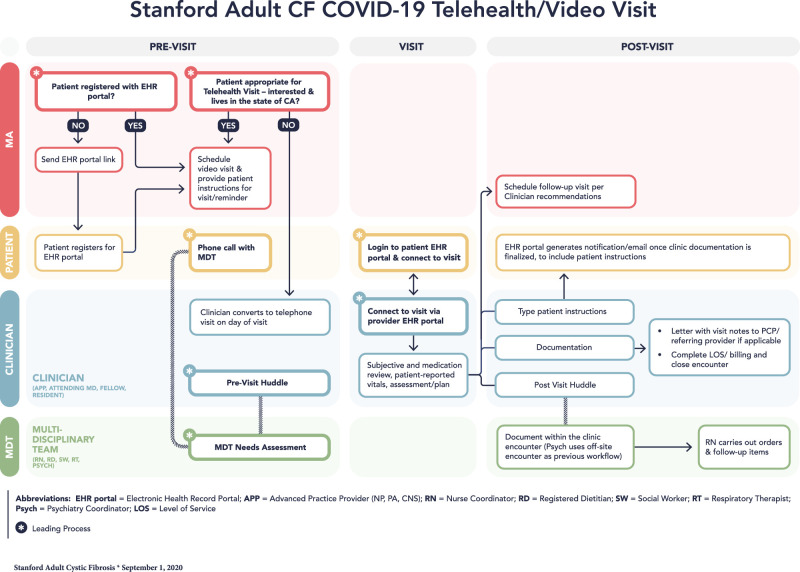
Stanford adult cystic fibrosis COVID-19 telehealth/video visit functional model.

The Stanford Health Care (SHC) Adult CF program serves approximately 250 patients aged 18 years and older residing in the surrounding metropolitan area, as well as Central and Southern California, with only 4% residing out of state. Patients are evaluated at 1- to 2-month intervals. After our institution’s expansion of its existing telemedicine capabilities, our team met weekly to develop a video visit functional model (Figure 1), using the Plan, Do, Study, Act (PDSA) improvement strategy.^17^ This project was exempt from Investigational Review Board approval as it met requirements for clinical quality improvement.

All staff completed a competency module outlining provider and patient requirements for the video EHR portal. To ensure HIPAA compliance, providers were required to utilize institutional computers from a private space. A letter was sent to all center patients via EHR bulk messaging notifying them of the transition to telemedicine along with access instructions. Medical Assistants were responsible for changing in-person visits to video visits, ensuring patient login access to the EHR, as well as scheduling future video visits. Patients connected to the visit via a smartphone or a computer. Our Advanced Practice Providers (APPs) partnered with nurse coordinators to triage which patients required urgent in-person care. Patients received a phone call from nurse coordinators 1 day prior to their visit to confirm their appointment.

A clinician (physician, APP) led the visit and performed a CF-focused review of systems, medication reconciliation, and assessment incorporating patient-provided vital signs. A physical examination limited to inspection was performed. Documentation mirrored an in-person note including patient consent to the risks, benefits, and limitations of receiving care virtually. Given the constraint of single-provider access to the video portal, the multidisciplinary team (MDT) composed of registered nurse coordinators, registered dietitian, social worker, respiratory therapist, and psychiatrist provided their services via telephone calls before or after the scheduled video visit. The clinician billed at parity with in-person visits based on time or medical complexity. Technical issues required converting the visit to a telephone encounter, with billing and documentation remaining unchanged. For sick visits, the clinician determined if symptoms were manageable with outpatient therapies. For in-person evaluation, the patient was directed to the Emergency Department, where rigorous COVID-19 screening, testing, and necessary isolation were implemented.^18^

To maintain communication and collaboration between the clinicians and the MDT, pre- and postclinic team meetings were held remotely using a secure video conference link. The previsit huddle included review of patients’ recent health changes and anticipated needs; the postvisit debriefs communicated pertinent patient findings.

**Figure 2.**
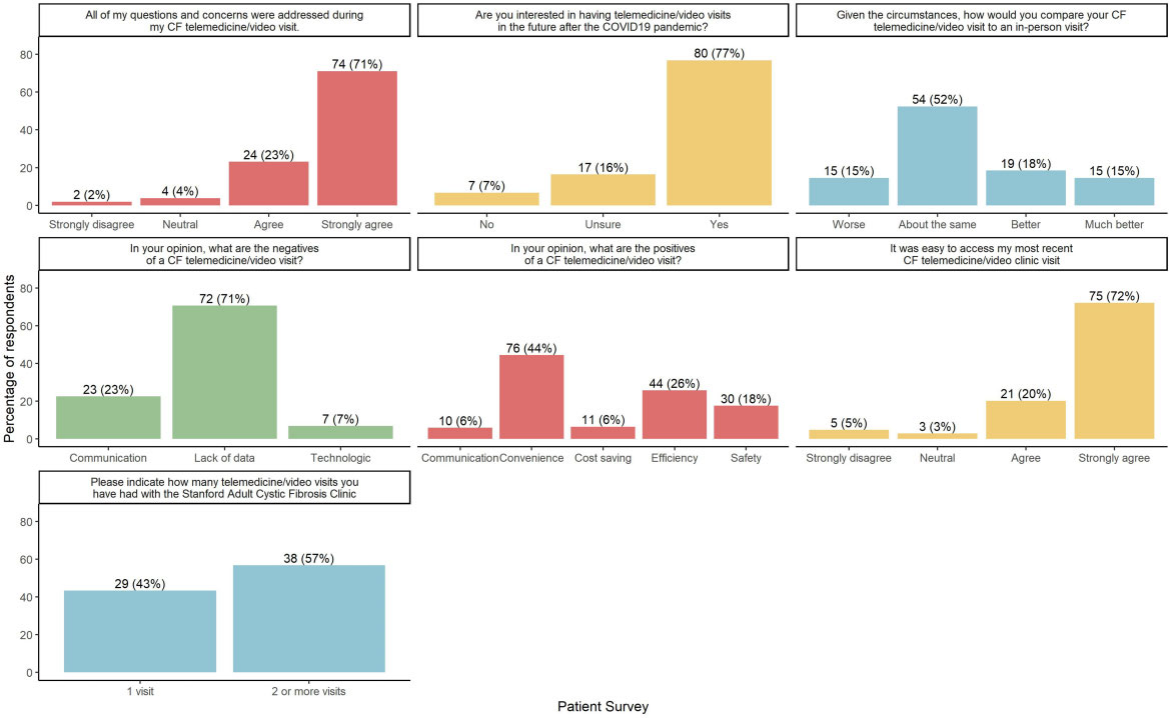
Stanford adult cystic fibrosis COVID-19 telehealth/video visit patient survey.

**Figure 3.**
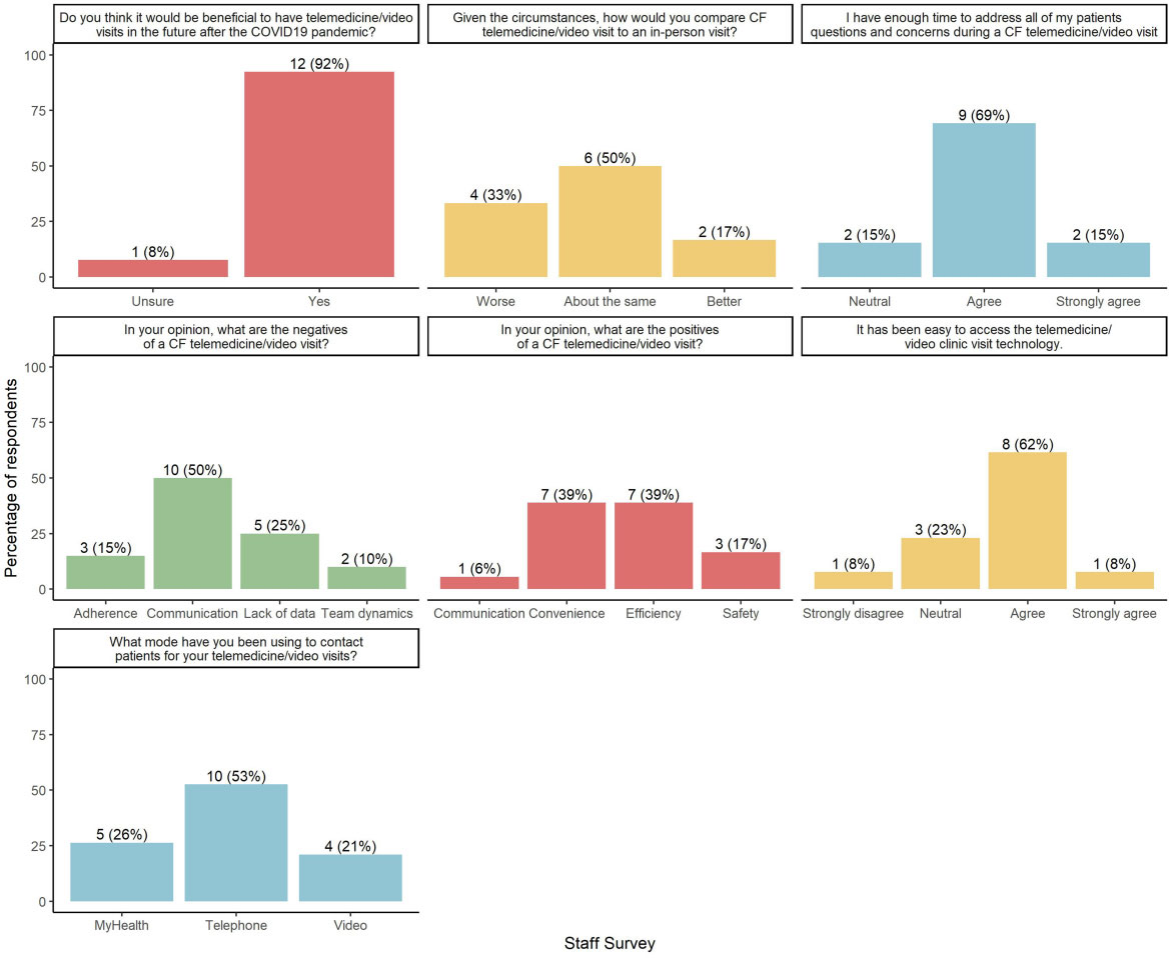
Stanford adult cystic fibrosis COVID-19 telehealth/video visit staff survey.

To evaluate this model, we developed and administered anonymous satisfaction surveys to patients and staff over an 11-week period between March and May 2020. They included multiple-choice and open-ended questions designed in SurveyMonkey. A survey link was included in the after-visit instructions accessed by the patient via the EHR portal. Staff received a link in their institutional email. The narrative responses were aggregated into broad categories (Figures 2 and 3).

## RESULTS

Of 245 patient survey links administered, we received 104 responses (42% response rate). Although specific demographic data were not included in our survey, our overall center demographics were notable for 96% in-state and 4% out-of-state. Of the respondents, 92% reported ease of access to the visit and 94% expressed that their questions and concerns were addressed. Most patients (52%) agreed video visits were comparable to an in-person visit and 77% were in favor of continuing this modality outside of the pandemic. Positive narratives described communication, convenience, cost savings, efficiency, and safety, with convenience ranking highest followed by visit efficiency. Safety narratives that addressed the decreased risk of exposure to COVID-19 infection were a minimally reported concern by patients. Negative narratives highlighted communication, technologic issues, and lack of diagnostic data, with the latter as the most frequently mentioned drawback, given the lack of spirometry and sputum collection (Figure 2).

Staff survey response rate was 100%. The majority reported ease of access to the telemedicine technology (69%) and adequate time to address patients’ questions and concerns (85%). Staff contacted patients by telephone (77%), followed by EHR messaging (38%) and video visits (31%); the lower distribution of video utilization is due to the single-provider capability (clinician) of this technology at the time of our survey. Most staff perceived telemedicine as comparable to in-person visits and felt that continued video visits after the pandemic would be beneficial. Positive narratives addressed communication, convenience, efficiency, and safety, with convenience and efficiency ranking the highest. Negative narrative categories included adherence, communication, lack of diagnostic data, team dynamics, and technology. Communication and lack of diagnostic data were the most common responses (Figure 3).

## CONCLUSION

Perceptions of this telemedicine care model by our patients and team revealed that video visits were convenient, efficacious, and comparable to in-person visits, with interest for its utilization beyond the pandemic era. Lack of spirometry and sputum collection was perceived concerns of patients and staff. We are in the process of integrating home spirometers and home sputum collection kits to address these barriers.

While nothing can replace an in-person visit to promote a therapeutic relationship, telemedicine has preserved CF care delivery during the COVID-19 pandemic. The unknown landscape of COVID-19 viral mutations will continue to challenge the delivery of healthcare. Our detailed telemedicine functional model serves as a foundation to navigate this modality, while we continue to collaborate with the CF community at large to identify key drivers to overcome its barriers. Further research is required to determine the safety, effectiveness, and impact of this modality compared with the traditional CF care model.

## Funding

This project received no specific grant from any funding agency in the public, commercial, or not-for-profit sectors.

## AUTHOR CONTRIBUTIONS

All authors who contributed to this article, and who are listed meet all four criteria for authorship according to the ICMJE guidelines for authorship.
